# Comparison of Prestressing Methods with CFRP and SMA Materials in Flexurally Strengthened RC Members

**DOI:** 10.3390/ma15031231

**Published:** 2022-02-07

**Authors:** Janusz Rogowski, Renata Kotynia

**Affiliations:** Department of Concrete Structures, Lodz University of Technology, Al. Politechniki 6, 93-590 Lodz, Poland; renata.kotynia@p.lodz.pl

**Keywords:** prestressing, flexural strengthening, SMA, CFRP, RC beam, comparison

## Abstract

Over the years, prestressing concrete has become a well-known technique to improve the ultimate and serviceability state of RC members. Besides steel reinforcement, relatively new materials such as carbon fiber reinforced polymers (CFRP) or especially shape memory alloys (SMA) can be used to active strengthening. The main scope of this paper is to compare various prestressing methods using carbon composites and memory steel alloys. A description of SMA, shape memory effect, its utilization for prestressing, and CFRP materials are presented in the paper. Moreover, current state-of-the-art developments in the field of both materials, considering prestressing systems and available anchorage, material behavior, creep and stress relaxation, durability issues, thermal compatibility with concrete, and fire behavior, are described. A general revision of previous studies based on flexural strengthening using both materials is conducted and the selected results of these studies are briefly presented. The behavior of RC beams after strengthening with mentioned techniques is compared and discussed. Selected on-site applications are described to confirm the feasibility and practicality of the strengthening systems. Finally, the main advantages and disadvantages of CFRP and SMA materials for prestressing concrete structures are summarized and further recommendations for the future research are listed.

## 1. Introduction

Plenty of existing structures require retrofitting and strengthening. This is most often important due to the deterioration of structures, changes in using their functionality, or increasing design requirements. In the case of concrete structures in Poland, traditional methods of strengthening are still the most popular. These methods of retrofitting (with traditional materials such as concrete or steel) are well known and described by Al-Mahaidi et al. [1]. However, these techniques take up a lot of extra space, require unloading of the structure, and the strengthening efficiency will be significant after reloading. They are classified as passive strengthening techniques and do not affect significantly the serviceability limit state. To avoid these limitations, carbon fiber reinforced polymer (CFRP) composites have been used for over thirty years all over the world [2,3,4,5] and for over twenty years in Poland [6,7]. These materials exhibit great mechanical characteristics and high durability. A description of CFRP materials is presented in Section 3.

Strengthening with prestressed CFRP materials requires a mechanical anchorage that properly transfers prestressing force to the strengthened member to avoid debonding of composite material from the concrete surface. The anchorage systems are highly complex due to additional devices based on the hydraulic jacks, which are used for prestressing the CFRP laminates [8,9,10,11]. Due to the mentioned parameters, the overall costs of strengthening with CFRP materials increases significantly. The prestressing CFRP systems are described in detail in Section 4.1.

A promising alternative for the prestressing methods of existing concrete members, which substantially reduces these expenses, is prestressing using shape memory alloys (SMA). Several research studies about the most popular nickel-titanium based alloys (NiTiSMA), have been tested for decades and confirmed their capabilities for retrofitting concrete members [12,13]. However, these applications in the civil engineering industry have been limited due to their high material costs. Another type of SMA materials, based on iron and called FeSMA, with a reasonable production cost (in comparison to the NiTiSMA) has been developed for the last years and applied in 2009 [14]. More details referring to SMA materials are described in Section 2. These methods of strengthening concrete structures with prestressed CFRP and FeSMA gave promising opportunities not only for many existing structures but also for new structures. It seems reasonable that these methods should be compared in many studies. Hosseini et al. [15] conducted complex research for metallic structures. However, the number of research projects in this field is still limited for concrete structures.

This paper aims to present the current state-of-the-art developments in the topic of flexural strengthening of RC structures with prestressed SMA materials in comparison to that with CFRP. This comparison contains an analysis of materials behavior, available prestressing systems, and previous studies with applications of both memory steel and carbon composite materials. The paper provides a background for further research in this field which can be of significant interest. 

## 2. Description of Shape Memory Alloy Materials

**Figure 1:**
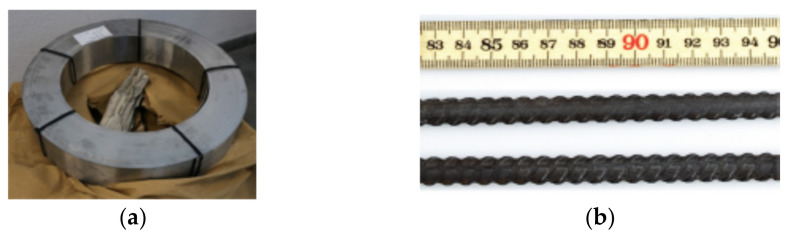
Iron-based shape memory alloy in the form of: (**a**) strips, reprinted with permission from ref. [18]. Copyright 2018 Elsevier; (**b**) bars, reprinted with permission from ref. [20]. Copyright 2016 Elsevier.

Shape memory alloys are metallic alloys (based on nickel, copper or iron) that have a unique property which allows them to return to their initial shape after having been permanently deformed. Examples of FeSMA materials are presented in Figure 1. The flexural strengthening of RC members with prestressed FeSMA strips has been widely tested in published research [16,17,18] and the first on-site Swiss application in carpentry used the FeSMA strips to the active strengthening of the RC slab [19]. Memory steel bars were embedded in a shotcrete layer [20] or applied as near-surface mounted reinforcement [21,22,23,24].

**Figure 2:**
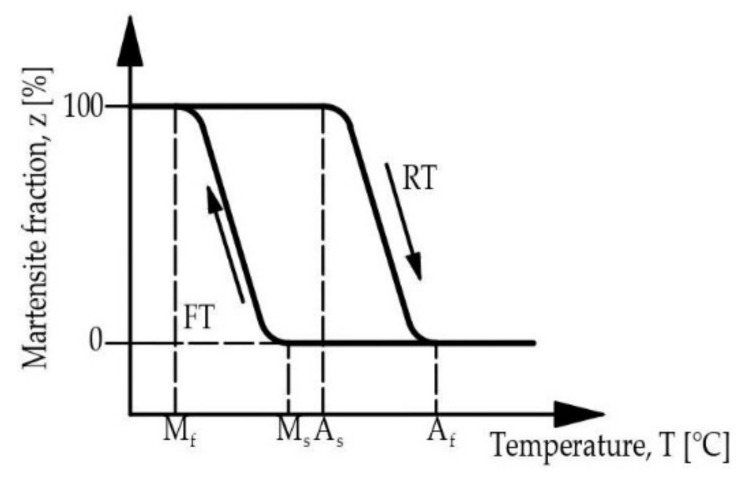
Temperature characteristics of the forward and reverse transformation (FT and RT, respectively), republished from [26].

Change of deformability is called the shape memory effect (SME) and has to be activated by heating [18]. If the return to the initial shape occurs automatically by unloading, the effect is named superelasticity or pseudoelasticity [13,25]. SME is connected with (reversible) phase transformation of the lattice structure of the alloy [26]. It consists of a transformation between the austenitic state (high-temperature phase with a regular cubic crystal structure) and the martensitic state (low-temperature state with an irregular crystal structure) [21]. The first phase transformation is called a martensitic (or forward) transformation (FT) and appears when the material is cooled (in absence of stresses). The martensite begins to appear at the temperature, M_s_, (martensite start) and the process finishes at the temperature, M_f_, (martensite finish). The reverse transformation (RT) is induced by heating the material (in absence of stresses), begins at temperature known as, A_s_, (austenite start) and has the end at the temperature, A_f_, (austenite finish) [27]. These processes are presented in Figure 2. What is important is that they do not occur at the same temperature (thermal hysteresis takes place) [26].

Depending on the ambient and transformation temperatures profile of the alloy material, different phenomena occur in the material [13,20]. It can be assumed that the ambient temperature for outside applications is between −20 °C (in winter) and +60 °C (in case of strong solar radiation). Various kinds of SMAs can have significantly different transformation temperatures. These temperatures are not only related to the composition of the alloy but also the thermomechanical treatment during production [26,27]. Moreover, they may change if the material is under mechanical load, which is affected by a value and a way of loading [13].

**Figure 3:**
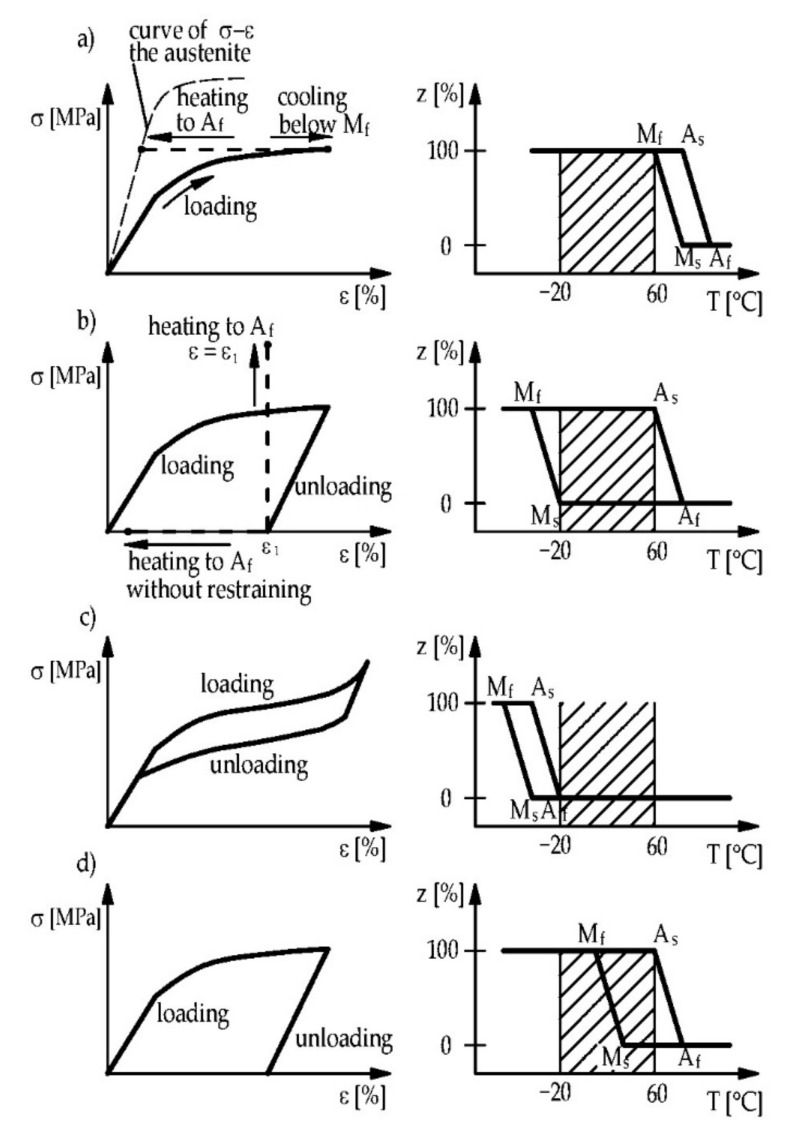
Stress-strain curves under loading (**left**) and transformation temperature profiles without loading (**right**): (**a**) strain and stiffness changes against a constant force; (**b**) shape memory effect; (**c**) superelasticity; (**d**) martensitic damping. Reprinted with permission from ref. [13]. Copyright 2005 Springer Nature.

Typical material phenomena and their associated temperatures profiles are presented in Figure 3. Alloys, for which martensite is a stable phase at ambient temperature, undergo pseudoplastic deformations if the yield strength is exceeded. If the temperature increases above A_s_, the reverse transformation begins. As strain-stress curves for martensite and austenite are different, a change of stiffness takes place. An increase in the stiffness of the alloy causes the strain decreases against a constant force (Figure 3a). This phenomenon can be used for many applications (such as for actuators) [13].

**Figure 4:**
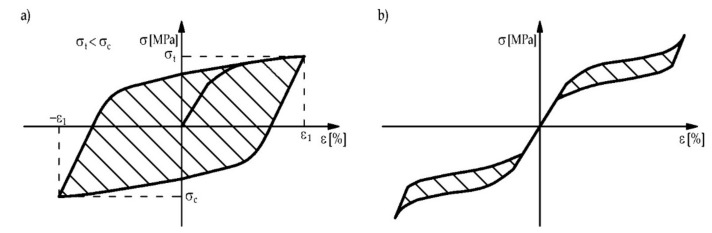
Stress-strain curve of alloy: (**a**) for stable martensite and cyclic loading and (**b**) for stable austenite and superelasticity alloy behavior (Reprinted with permission from ref. [13]. Copyright 2005 Springer Nature).

Another property of the alloy, if martensite is the stable phase, is the ability to dissipate energy. This phenomenon is called martensitic damping and occurs when the material is subjected to cyclic loading (Figure 4a). A part of the energy can be dissipated due to a difference between the loading and unloading path (Figure 3d) [13].

If austenite is the stable phase at ambient temperature, external loading will induce martensite transformation (without change of temperature). The reverse transformation occurs automatically after unloading. This phenomenon is called superelasticity or pseudoelasticity and it exhibits some stress-strain hysteresis [27], as shown in Figure 3c. The amount of dissipated energy corresponds to the area between the loading and unloading path (see Figure 4b [13]). More details about the damping properties of SMA can be found in other publications [28,29,30].

**Figure 5:**
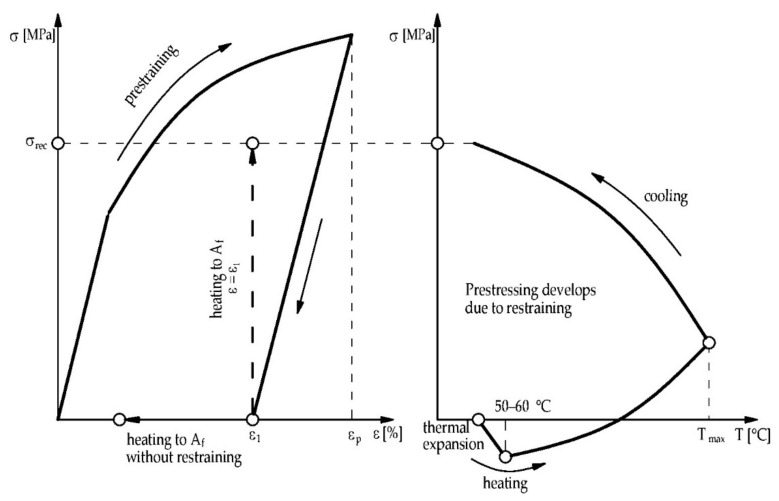
Characteristics of prestraining and activation SMA materials (Reprinted with permission from ref. [19]. Copyright 2018 John Wiley & Sons-Books).

Permanently deformed alloys in the martensitic state can return to their initial shape after heating above the temperature A_f_. This phenomenon is called the one-way shape memory effect or pseudoplasticity [13]. If the deformed material is restrained, some mechanical stress occurs after heating in the material (Figure 3b). In the beginning, due to the thermal expansion of the alloy, reduction of stress can be observed in Figure 5. However, the SME starts taking place after a certain temperature threshold. If the temperature grows to the peak temperature T_max_, tensile stress develops in the material. The stress obtained by cooling the specimen down is referred to as “recovery stress” (σ_rec_, see Figure 5) and can be used for prestressing some elements of civil engineering structures [19].

**Figure 6:**
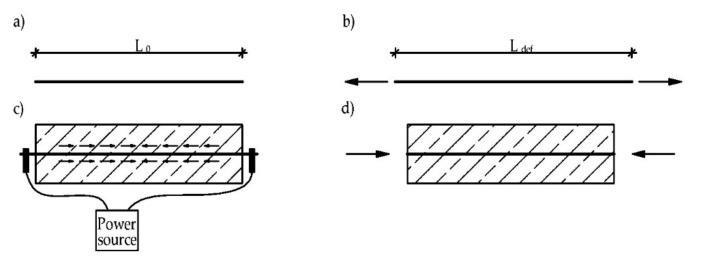
Scheme of prestressing of concrete member with SMA reinforcement: (**a**) initial SMA element with length L_0_; (**b**) prestrained element with length L_def_; (**c**) heating of the SMA, e.g., with electrical current; (**d**) concrete is prestressed, republished from [31].

The method of prestressing concrete structures using SMA reinforcements is shown in Figure 6. A strip, bar, or wire of SMA is prestrained (permanently elongated), then embedded (or mounted) in concrete. If the SMA reinforcement is heated (after curing of concrete) and its deformations are restrained, the recovery stress develops in the SMA. It causes some compressive stress to occur in the concrete. Different reinforced concrete members can be prestressed using this effect [31]. This method of prestressing does not require hydraulic jacks, ducts, or an anchor head. In the case of existing structures, it is sufficient to use end-anchorage (e.g., a direct fastening system) [32]. Moreover, the strongly curved structures can be also reinforced with this technique since no prestress force loss due to friction takes place [33].

## 3. Description of Carbon Fiber Reinforced Polymers

Fiber-reinforced polymers (FRPs) are composite materials composed of at least two components, namely fibers and a polymer matrix. The matrix performs the role of binder, distributes the uniform load, and protects the fibers against environmental effects [34]. The fibers have excellent mechanical properties and strength in tension, and therefore they are effective reinforcement materials [35]. From a structural point of view, the most promising material are carbon fibers (due to the highest value of Young modulus in comparison with other types of fibers) [34]. Hence, carbon fiber reinforced polymers (CFRPs) are selected to be compared with SMA and are briefly described in this section.

**Figure 7:**
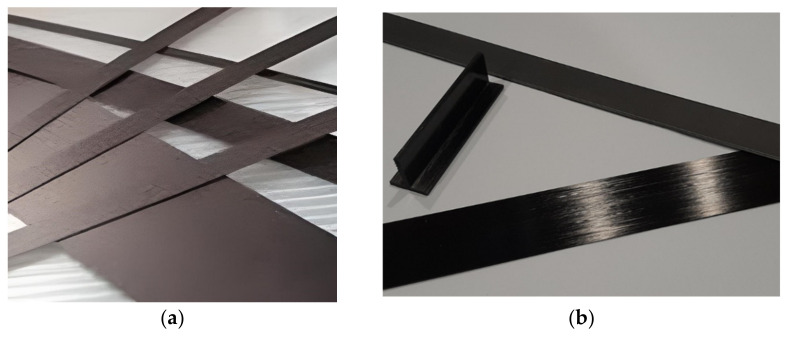
Examples of CFRP materials: (**a**) strips; (**b**) strips and T-shape profile.

The properties of CFRPs depend on the volume of each component and the orientation of fibers. CFRPs are available for strengthening of civil engineering structures in the form of [34,35]:

- Unidirectional pultruded laminates (strips)—Figure 7a,b;
- Sheet (with fibers in one direction) or fabrics (fibers are arranged in at least two directions) that are usually impregnated in-situ;
- Rods of bars that are made by pultrusion;
- Profiles (T-shape, L-shape)—Figure 7b.

The first application of CFRP materials was in the United States at the California Department of Transportation, Caltrans [36]. Application of CFRP materials for flexural strengthening of RC structures started in the 1980s at the Swiss Federal Laboratory for Materials Testing and Research (EMPA) [37,38]. Polish experiences in structural strengthening started from bridges with the first application of CFRP laminates in 1992 on the bridge over the Wiar river [39]. One year later, the second application with combined CFRP laminates and sheets was performed on the bridge over the Bystry canal [40]. Other Polish CFRP applications on the RC structures were published in [6,10,41,42]. Much more effective flexural strengthening with prestressed laminates was carried out in [7,43,44,45,46,47].

Most research and field applications on the flexural strengthening of RC members were carried out on simply supported beams and slabs strengthened on the bottom surface of the RC members without additional anchorage in the support region.

**Figure 8:**
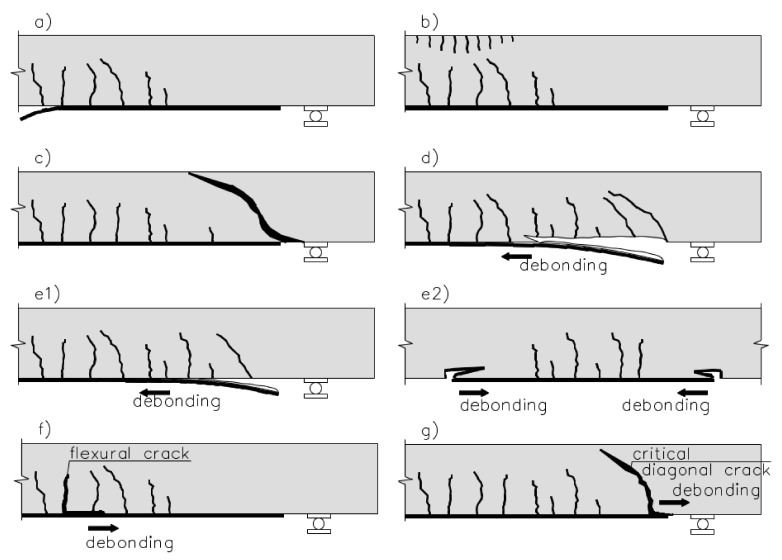
Failure modes of FRP-plated RC beams: (**a**) FRP rupture (R); (**b**) concrete crushing (CC); (**c**) shear failure; (**d**) concrete cover separation (CCS); (**e1**) plate end interfacial debonding (PE); (**e2**) plate end debonding in too short laminates; (**f**) intermediate flexural/shear crack-induced interfacial debonding (IC); (**g**) critical diagonal shear crack-induced debonding (CDC), Reprinted with permission from ref. [49]. Copyright 2002 Elsevier.

The existing research on reinforced concrete members flexurally strengthened with FRP materials can fail in several different ways, which are completely different in comparison with original RC members. There is a wide literature referring to classification of the failure modes published for the last two decades. The most common classification based on test results of the existing research was presented in [48]. Eight categories referring to material failure and interface debonding failure modes are summarized in Figure 8.

The most common failure mode is debonding of the FRP laminate from the concrete surface which may proceed as intermediate crack induced interfacial debonding (ICD) initiates at the flexural/flexural-shear cracks in the highest bending moment region (Figure 8f) and propagates by gradual debonding of the laminate from the flexural crack to the end of the FRP end (Figure 8f,g).

When debonding occurs at or near the end of a laminate it may proceed in three different ways:

- Critical diagonal crack (CDC) debonding occurs after the formation of a major shear crack intersecting the plate near its end and develops along with the laminate-concrete interface to the plate end (Figure 8b);
- Concrete cover separation (CCS) (Figure 8c);
- Plate end interfacial debonding (PEI) (Figure 8d).

Two additional failure modes refer to the FRP rupture and concrete crushing. However, the second one is possible only for the RC members of low concrete strength and high reinforcement ratio.

The effectiveness of the flexural strengthening depends on several factors including FRP type; axial stiffness and the number of CFRP layers; a distance of the CFRP end from the support; the existing longitudinal and shear steel reinforcement ratio; and bending moment distribution. Although EBR CFRPs increase the load-bearing capacity of an RC member, they do not significantly change the cracking load and deflections under the service loads. To gain the greatest advantage of the EBR technique, CFRP prestressing has been proposed to improve the serviceability of strengthened structures in order to reduce crack widths effectively, to relieve stress in the internal reinforcement, to enable control the crack distribution, limit deflection, and to increase the stiffness and the load capacity of RC members.

## 4. Comparison of Prestressed FeSMA and CFRP Behavior

This section is focused on a comparison of CFRP and SMA behavior and techniques of their applications to the strengthening of RC structures. The important parameters considered in this analysis contain tensile strength, Young’s modulus, creep and relaxation, thermal compatibility, durability, behavior at elevated temperatures, prestressing procedure, prestressing force, and anchorage systems.

Memory steel based on iron has reasonable production cost, higher elastic modulus, and lower activation temperature in comparison with other types of SMA materials [50]. FeSMA materials described in Section 1 are chosen as a promising alternative for prestressing instead of CFRP materials.

### 4.1. Prestressing and Anchorage Systems

The state-of-the-art strengthening with prestressed FRP materials was published in [9,34,51,52,53]. Three main CFRP prestressing techniques were published in [9]:

- Externally applied reinforcement (EAR) which include both externally bonded reinforcement (EBR) and unbonded reinforcement (without adhesive between composite and concrete); the unbonded reinforcement system is much less popular, but the applications can be found in [47,54];
- Near-surface mounted reinforcement (NSMR) [55,56,57,58,59,60,61];
- Externally post-tensioned (EPT).

In the EAR technique, CFRP reinforcement is in most cases prestressed to reduce existing deflections and to extend the flexural capacity of the existing concrete structures (direct prestressing method [53]). CFRP prestressing requires using a hydraulic jack which must be initially fixated on the concrete surface. In most cases, these elements are temporarily mounted and after strengthening they are removed [52]. The majority of CFRP prestressing systems requires also mechanical anchorage (MA) at both CFRP ends. Various types of MA systems have been investigated over the years and have been summarized in [8]. The most popular prestressing and anchorage commercially available systems have been developed by: S&P Clever Reinforcement Company AG, Seewen, Switzerland [62]; “Leoba-CarboDur” system by Leonhardt, Andrä und Partner, Stuttgart, Germany [63]; “Stresshead” system by Sika Bau AG, Zurich, Switzerland and Stress Head AG, Luzern, Switzerland [54]; “gradient—anchored” prestressing system by [64,65,66]; TENROC “gradient—anchored” prestressing system by Tenroc Technologies, Gothenburg, Sweden [67]; Polish Neoxe Prestressing System [46] and NPS II [43] by Neoxe, Cracow, Poland. Several noncommercial systems were used in the research: multi-layer CFRP sheets technique [55,68]; mechanically anchored, CFRP anchored U-wraps sheets [51,69]. Prestressing of CFRP materials maximizes the utilization of composites, excluding brittle failure modes caused by debonding [70,71]. For this reason, the system of strengthening with pretensioned laminates requires mechanical anchorage of their ends in the concrete surface [72].

In most cases, the MA systems are based on anchor plates. A direct prestressing system consists of the passive anchorage (dead end), while the second end is the active one [53,72] which can be additionally combined with the tensioning device [11]. The only non-mechanical anchorage system known as the “gradient-anchorage” (GA) was developed by Meier at EMPA [64,66] with the first field application in Poland [73]. The study on different anchorage systems published by Correira et al. [74] indicated that the MA system prevented premature debonding failure.

Reinforced concrete beams strengthened with prestressed CFRP laminates show three groups of failure modes: under-reinforced RC members failed due to FRP rupture (R); over-reinforced RC members with composite reinforcement applied excessively, resulting in concrete crushing (CC). However, this failure mode is possible only for the RC members characterized by low concrete strength and high reinforcement ratio; intermediate crack induced by interfacial debonding (ICD), which initiates at the flexural/flexural-shear cracks in the highest bending moment region and propagates by gradual debonding of the laminate from the flexural crack to the FRP end and the group of RC members with the reinforcement not anchored sufficiently, in which leads debonding of FRP ends (plate end debonding (PE), concrete cover separation (CCS), anchorage failure (AF)) published in [48,64,66,72,75,76,77,78].

The parameters affecting strengthening efficiency with externally bonded FRP prestressed materials contain type of FRP material (laminate, sheet); FRP stiffness; existing flexural tensile reinforcement ratio; existing shear reinforcement ratio; stiffness of the strengthened RC member (slab, beam); the size of the strengthened RC member; type of strengthening system (mechanically anchored, fully efficient (FRP rupture) or partially efficient (FRP sliding from the anchored system) and preloading level.

The flexural strengthening of RC members strengthened with pretensioned NSM FRP stirps or bars confirms the much higher efficiency of this technique compared to the passive strengthening. The main effect of pretensioning is based on the higher utilization of the tensile strength of the FRP materials, which contributes to the higher load-bearing capacity under service and ultimate load [55,56,57]. The effectiveness of the NSM method significantly depends on several factors including pretensioning level, cross-section and stiffness of FRP materials, and internal steel reinforcement ratio. Prestressing leads to an increase of the load-carrying capacity at concrete cracking and steel yielding; reduction of dead load deflections; reduction of cracks; and increase in the shear capacity by longitudinal prestressing [58,59,60,61].

The prestressing procedure for all types of SMA reinforcement is quite similar and it does not require using the hydraulic jacks. In the beginning, the SMA materials are initially prestrained at room temperature, and then the reinforcement (with permanent deformation) is applied and fixed to the concrete surface and the activation of the alloy is conducted by heating to the required temperature. The heat supply can be provided by infrared heating, electrical resistive heating [79] or using a gas burning torch [21]. Due to restraining the recovery stress develops in the SMA material (see Section 4.3). The restraining mechanism depends on the type of SMA and the strengthening technique [79]:

- For externally applied strips, the direct fastening system is used [80]. This method is based on the pre-drilling of the SMA strip to the concrete substrate and installation of the nails in the holes using powder-activated tools [19,79];
- The NSM strips or bars can be inserted into the concrete grooves which are filled with cement-based grout [17,24,81]. The bars can be also fixed using end-anchors [21,82];
- The NSM bars can be also embedded in the shotcrete layer [20,24].

### 4.2. Comparison of CFRP and SMA Tensile Behaviour, Creep and Relaxation

**Figure 9:**
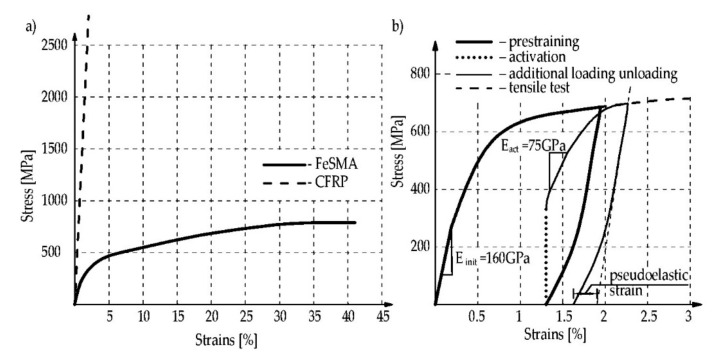
Stress-strain curves of FeSMA and CFRP materials: (**a**) under loading; (**b**) behavior of FeSMA at various stages (Reprinted with permission from ref. [18]. Copyright 2018 Elsevier).

FeSMA materials exhibit a similar stress-strain response as steel. The memory steel properties are affected by the alloy composite and the thermomechanical treatment during the production process [26]. In general, they have the higher ultimate strength (680–1000 MPa) and comparable or slightly higher ductility (ultimate tensile strain equal to 16–50%) to the steel [18,83]. They are isotropic materials in contrast to composites, which are fully anisotropic with various tensile characteristics in different directions [34]. The properties of composites materials depend on fibers, matrix type and volume fraction. The CFRP materials have three times higher tensile strength than FeSMAs but they exhibit ten times lower ductility in the ultimate tensile strain compared with FeSMA materials [84]. The mechanical properties of the chosen iron-based SMAs, CFRP strips and steel are summarized in Table 1 and presented in Figure 9a.

The initial elastic modulus of FeSMA is similar to low modulus CFRP and is lower than steel and the high modulus CFRP strips. It should be mentioned that Young’s modulus of the alloy depends on its actual state, which is about two times lower for the material after activation, E_act_, than for the material in elastic state, E_init_, see Figure 9b [18]. The behavior of SMA during unloading is nonlinear. A pseudoelastic strain is defined as the strain deviation from the linear elastic behavior and is presented in Figure 9b [18].

The creep behavior of FRP materials was investigated by Goertzen et al. [85] in the tensile creep research. No failure due to creep rupture was observed in the short-term (1600 h) at loads up to 77% of the ultimate tensile strength. The extrapolation of these data indicated that the tensile failure did not occur during a reasonable lifetime at the load corresponding to 65% of the tensile CFRP strength [85]. Hence, it is recommended to limit the prestressing level of carbon composites to 65% of the ultimate strength [86]. A negligible creep of the CFRP materials was confirmed in the studies [87,88] referring to CFRP rebars [89], sheets [87], and tendons [90], which demonstrated no significant stress relaxation in the long-term behavior of the CFRP composites. Prestress losses in the RC members strengthened with prestressed CFRP occurs in the first 100 h while a further decrease in the prestressing stress is negligible [91].

**Figure 10:**
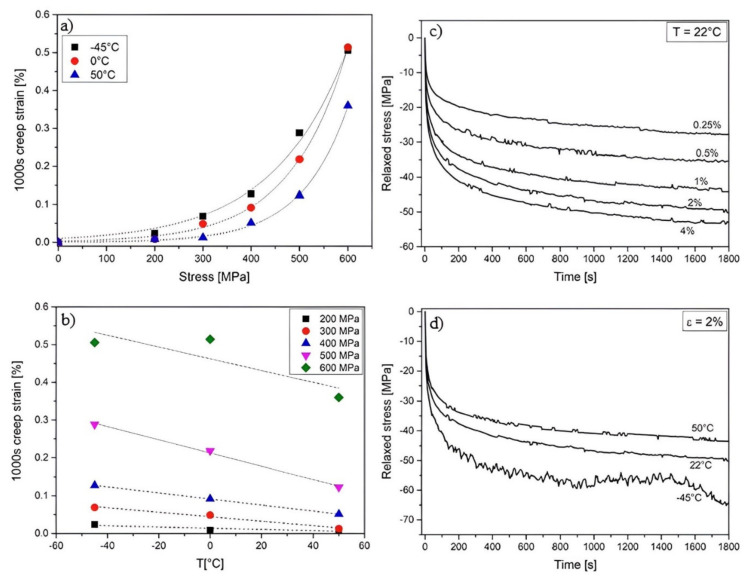
Creep and relaxation in SMA material: (**a**) creep strain after 1000 s as a function of constant stress; (**b**) creep strain after 1000 s as a function of temperature; (**c**) stress relaxation at room temperature for various constant strains; (**d**) stress relaxation at different temperatures for the strain of 2%; Reprinted with permission from ref. [96]. Copyright 2016 Elsevier.

A mount of tests referring to FeSMA relaxation has been carried out over the last years published in [18,92,93,94,95,96]. Both the stress relaxation at the constant strain and creep at the constant stress occurred in the SMA material. The study conducted by Leinenbach et al. [96] indicated that the creep strain was an order of magnitude higher than that for the high strength steels or Titanium (Ti) alloys. Moreover, for the temperature in the range from −45 °C to 50 °C, the creep and relaxation are greater with the temperature drop (see Figure 10) [96]. A recovery stress decrease equal to 10% and 20% was observed after 1000 h in [18,93,94], respectively. The greater prestress losses were affected by the SMA slip in the anchorage, temperature fluctuations and the live traffic loads. However, the total relaxation losses during the life cycle of the building can be estimated at 15% [15]. It should be mentioned that the second (or multiple) activations (re-heated to the activation temperature and cooled to the ambient temperature) of SMA can retrieve a significant part of the prestressing losses [95].

### 4.3. Achievable Prestressing Force

The prestressing force in the SMA technique depends on the recovery stress of the alloy which is obtained after activation. It highly depends on the alloy composition, thermo-mechanical training, level of prestraining, and activation temperature. Based on data from research conducted by Cladera et al. [26], it has been indicated that recovery stresses for different Fe-Mn-Si alloys (with various thermo-mechanical training) are in the range of 130–580 MPa. The tests conducted by Shahverdi et al. [18] indicated that the higher recovery stress can be reached by increasing the maximal temperature during activation (more phase transformation is induced by higher temperature). The recovery stress of 356, 389, and 445 MPa were obtained at the temperature of 177, 190, and 380 °C, respectively. It should be taken into account that the high temperature inside the concrete may lead to deteriorating its mechanical properties [26]. The recovery stress also increased with increasing the initial prestraining level with the plateau at 2% of prestraining. Based on the research published by Shahverdi et al. [18,97] it was indicated the optimal level of prestraining (at a temperature of 160 °C).

The prestressing level in CFRPs should be at least 25% and does not exceed 65% of the ultimate strength [98] (see Section 4.2). Prestressing equal to 50% of the CFRP tensile strength is suggested to be applied in the EAR technique due to technical and economic reasons [72,98,99]. However, 40% of the CFRP tensile strength is recommended for the NSMR technique [9]. In general, the recovery stress in memory steel is much smaller than the initial prestress in the CFRP reinforcement. Hence, in the case of a similar cross-section area of CFRP and SMA reinforcement, the prestressing force would be from two to four times higher for the CFRP prestressing.

### 4.4. Influence of Durability

Due to the non-metallic nature of CFRPs, these materials are fully corrosion-resistant. This is why environment exposure does not affect the decrease in the tensile CFRP strength [100,101].

**Figure 11:**
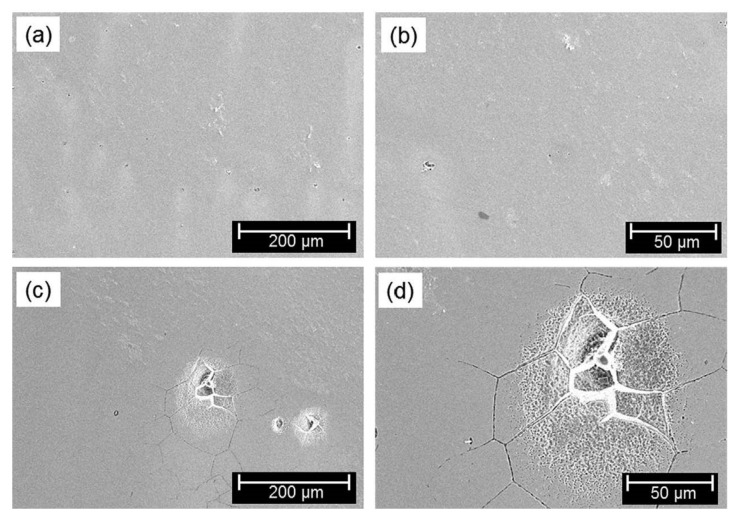
SEM images of corroded surfaces of SMA at pH 8.4: (**a**) without chloride, solution 15 mM NaHCO_3_ + 5 mM Na_2_CO_3_; (**b**) without chloride, the same solution, zoom in; (**c**) with chloride, solution 15 mM NaHCO_3_ + 5 mM Na_2_CO_3_ + 2.8 M NaCl; (**d**) with chloride, the same solution, zoom in (Reprinted with permission from ref. [102]. Copyright 2015 John Wiley & Sons-Book).

**Figure 12:**
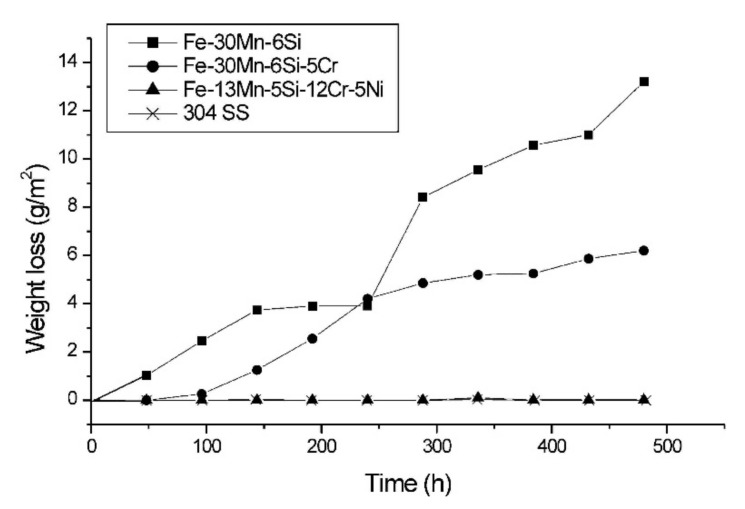
The weight loss in function of immersion time for different types of iron-based SMA and stainless-steel SUS 304 in a 3.5% NaCl solution (Reprinted with permission from ref. [108]. Copyright 2002 Elsevier).

The corrosion resistance of memory steel has been investigated for various iron-based SMAs in [102,103,104,105,106,107,108,109,110]. Based on the previous studies that are summarized in Table 2, it can be concluded that the smaller the exchange current density (i_corr_), the higher the corrosion resistance of the material in the tested solution. Lee et al. [102] and Joo et al. [110] indicated that iron-based SMA has better corrosion resistance in comparison with the conventional steel of type S500 and S400, respectively. The vulnerability to corrosion in chloride environment due to high manganese (Mn) content was identified in the study carried out by Rovere et al. [105]. Dias et al. [109] confirmed low corrosion resistance of the iron-based alloy regardless of different mechanical processing and heat treatment. Analysis of SEM images of the SMA surface after polarization tests at pH 8.4 in solutions without chloride (15 mM NaHCO_3_ + 5 mM Na_2_CO_3_) and with chloride (15 mM NaHCO_3_ + 5 mM Na_2_CO_3_ + 2.8 M NaCl) was conducted by Lee et al. [102]. As it can be seen in Figure 11, the pitting corrosion is visible in the solution with chloride while no corrosion pits are on the surface of the sample in the solution without chloride. It can be concluded that the SMAs are very sensitive to the chloride contamination of concrete since the chloride ions cause their local de-passivation which leads to corrosion pittings. As the high manganese content decrease the corrosion resistance of alloy, high chromium (Cr) and nickel (Ni) content in the alloy causes higher corrosion resistance of SMA [107,108], see Figure 12. The corrosion behavior in NaCl environment can be also slightly improved by the addition of copper (Cu) [103] and Lanthanum (La) [104] or a small amount of cerium (Ce) [106]. Nevertheless, advanced corrosion protection should be taken into account and applied for structures in environments with high chloride concentrations. The externally applied reinforcement can be protected by the reactive coatings and the embedment in the cementitious matrix in the case of the NSM FeSMA [111].

### 4.5. Thermal Expansion Coefficients

In the case of strengthening applications, proper thermal compatibility between the reinforced material and concrete is of great importance. A significant difference in the thermal expansion coefficients (CTE) imposes the additional stresses in the strengthened element that causes a decrease in the strengthening efficiency [112]. CTE of concrete is 10 × 10^−6^ K^−1^ [113] and the steel reinforcement is in the range of 11–13 × 10^−6^ K^−1^. The current study has demonstrated that the CTE of FeSMA is in the range of 14–16 × 10^−6^ K^−1^ that is closed to other austenitic steel materials [16,26,95]. The study conducted by Fritsch et al. [114] confirmed a small change in the prestressing level of the FeSMA strip (which was anchored to steel substrate) during the high-cycle fatigue tests as a result of daily temperature changes. Insignificant changes in the FeSMA strengthened girder were observed by Hosseini et al. [15] as a result of the daytime and nighttime temperature variations. However, CTE of CFRPs (along fiber’s direction) is close to 0 or even negative [34,115,116]. Hosseini et al. [95] demonstrated that the difference between the CTE of steel and the CFRP materials imposed the stress changes along with the CFRP plates due to the temperature changes during days and nights (the similar behavior for concrete with bonded CFRP can be expected). In another study by Hosseini et al. [117], the long-term measurement results showed that daily temperature changes can be the reason for the significant thermal-induced stress cycles in the non-prestressed bonded CFRP plate. Similar stress changes occurred in the prestressed unbonded CFRP plates. However, in this case, the stress was almost negligible compared to the existing CFRP prestressing level.

### 4.6. Behavior at Elevated Temperatures

**Figure 13:**
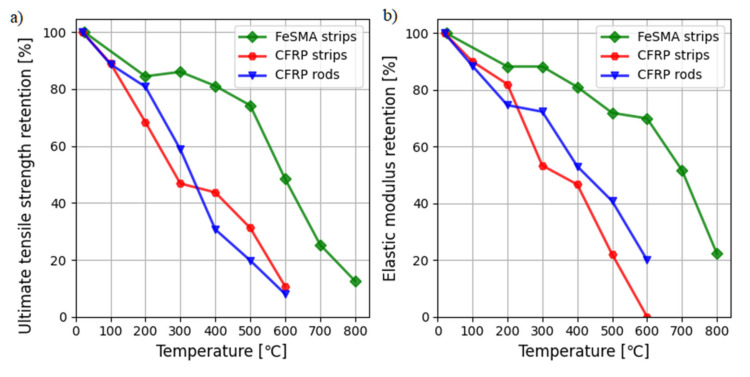
Parameters of FeSMA and CFRP materials at elevated temperatures based on the data given in [118,120]: (**a**) ultimate tensile strength retention; (**b**) elastic modulus retention.

The complex study on the structural fire behavior of the prestressed FeSMA was carried out by Ghafoori et al. [118]. The series of transient total deformation tests were performed on the FeSMA strips to determine the creep-onset and failure temperatures of the alloy at various service stresses (0, 80, and 240 MPa) and heating rates (5, 15, and 50 °C/min). The results of experimental investigations indicated that creep-onset temperatures were greater than 500 °C for all aforementioned conditions. The corresponding failure temperatures were approximately 70 °C higher than 500 °C. Moreover, the increase in the service stress caused a decrease in both the failure and creep-onset temperatures. The degradation of stiffness, yield, and ultimate strength of the memory steel subjected to elevated temperatures were similar to that of structural steel reinforcement investigated in [119]. The Young modulus and the ultimate strength of the FeSMA decreased by 20% with the temperature increase from ambient temperature to 400 °C (see Figure 13). The prestress losses increased with the temperature increase and the prestressing stress reduced to zero at about 320 °C. The prestress losses can be delayed by applying protective fire insulation materials.

The fire behavior of CFRP materials applied for strengthening concrete structures was investigated by Bisby et al. [121] and Firmo et al. [122]. These tests indicated that CFRPs subjected to elevated temperature suffer degradation in strength and stiffness [120,123,124]. The reduction in the ultimate strength and Young’s modulus is about 50% at 400 °C (see Figure 13). The most dangerous was FRP matrix degradation when exposed to the temperature of 300–400 °C, which caused releasing of heating and toxic gases [122,125]. Hence, it is necessary to provide proper fire protection not only due to CFRP degradation but due to smoke toxicity. However, conducted research [126,127] demonstrated the efficiency of thermal insulation to protect CFRP strengthened concrete members.

## 5. Literature Review

A limited number of research studies containing a comparison of flexural strengthening using CFRP and FeSMA materials have been conducted so far. The experiments and computational analysis based on literature [17,19,61,128,129,130,131] are summarized in Table 3. Other research studies dedicated to flexural strengthening using iron-based memory steel are summarized in Table 4. Finally, Table 5 presents selected research on flexural strengthening with prestressed CFRP materials.

Analysis of previous studies (mentioned in the above tables) revealed that different failure modes are exhibited for RC beams strengthened with FeSMA and CFRP materials. The majority of FeSMA beams failed due to concrete crushing after yielding steel (as unstrengthened reference beams) which is connected with their low longitudinal steel reinforcement ratio. In two prestressed beams [17], one of the FeSMA strips ruptured shortly before concrete was crushed. It should be highlighted that no anchorage failure was observed. The failure mode of beams strengthened with CFRP materials depends on factors such as the prestressing technique, prestressing level, anchorages, and adhesives. The most common failure mode was connected with debonding or rupture of the bar, strip, or rod. In some cases, anchorage failure or sliding of strip end from anchorage took place.

Selected results from the previous studies are presented in Table 6. Prestressing increased the cracking load and deflection. Strengthening with FeSMA materials allowed to keep the ductile behavior similar to unstrengthened RC beams with higher value of cracking, yielding and ultimate loads while the behavior of beams strengthened with CFRP was much more brittle with a significant reduction in ultimate deflection. However, the ultimate beam capacity was greater for beams with CFRP compared to that with memory steel.

To highlight the better ductility of the FeSMA strengthened beams compared to that strengthened with the CFRP reinforcement, the ductility index was introduced in Table 6. It was calculated as the ratio of mid-span deflections at ultimate and steel yielding states. The ductility index was much higher for the beams with FeSMAs. The yielding nature of the memory steel caused the higher deflections of RC beams before the concrete crushing.

Michels et al. [19] compared reinforced concrete beams with various types of strengthening: without strengthening, passive strengthening with bonded CFRP strips without end-anchorage and active strengthening with FeSMA strips. The FeSMA strips were fixed to the concrete with the direct fastening method that fully resisted the anchorage zones from debonding. The structural behavior of beams strengthened with FeSMA was more ductile than the beam with CFRP strips that failed due to CFRP debonding. The CFRP and concrete compressive strains were equal to 6.9‰ and 1.0‰, respectively, which confirmed only partial utilization of CFRP strips and concrete. However, the beams prestressed with FeSMA strips failed due to the concrete crushing with a maximal concrete strain equal to 2.5‰. For unbonded external prestressing tendon, more concentrated beam rotation occurred within the maximal bending moment zone with a slightly lower ultimate concrete compressive strain value [19,142]. Although all of the beams had similar crack spacing, the smaller length of crack distribution occurred in the beams strengthened with FeSMAs.

Five RC beams strengthened with NSM reinforcements and one reference beam without strengthening were analyzed by Shahverdi et al. in [17]. The beams had different types of strengthening: one beam reinforced with two non-activated FeSMA strips, three beams with two activated FeSMA strips and one beam strengthened by only one non-prestressed CFRP strip (to gain a similar strengthening effect). The FeSMA strips were only glued with cement-based mortar in grooves (without end anchorages) and their bond behavior was good. The prestressed beam exhibited much better performance in serviceability state (e.g., higher cracking load and smaller mid-span deflections (up to a load of ~12 kN)). However, prestressing does not increase the ultimate load in comparison to the non-prestressed beam with FeSMA which is similar behavior to the conventional prestressing. It was confirmed by Shahverdi et al. in [129] who carried out the nonlinear simulation based on the previous study and effects of various parameters (e.g., prestressing level, FeSMA or steel reinforcement ratio, compressive strength of concrete) on the structural behavior of NSM FeSMA beam were analyzed. The increase in ultimate load and deflection was observed when the concrete compressive strength increased but the cracking and yielding deflections and loads did not change significantly. The prestressing with NSM FeSMA strips was more efficient for beams with a low steel reinforcement ratio.

A study by Raad and Parvin [130] presents finite element analysis of the flexural strengthening of RC beams using NSM CFRP, GFRP and SMA rods. Moreover, several beams were strengthened with the hybrid (both CFRP and GFRP) or coupled CFRP-FeSMA rods. The effects of different prestressing levels (0%, 20%, 30%, and 40% of the ultimate strength) were investigated. The behavior of both CFRP and FeSMA strengthened beams was similar for all prestressing levels before cracking. Higher prestressing level caused slightly higher cracking load and higher yielding load for both materials. The steel yielding and the ultimate loads of the beams strengthened with FeSMAs were slightly lower (by 11–16% and 13–20%, respectively) in comparison with the CFRP strengthened beams, but they had a significantly higher increase in the ultimate deflection (by about 50%).

A numerical comparison of RC beams strengthened with the NSM techniques was conducted in [131]. One reference beam, two beams reinforced using prestressed CFRP rod and two beams with activated FeSMA strips were analyzed. Two different prestressing forces (28 and 56 kN) for each prestressing method were used, but different ways were used to gain these effects. The number of strips was twice higher in the case of the FeSMA beam, while the CFRP strips were prestressed with the higher initial strain (FeSMA_Beam2 and CFRP_Beam2, respectively; Table 3). These parameters led to the higher axial stiffness of the FeSMA beam and resulted in the higher ultimate load. However, all of the beams with the additional reinforcement exhibited a comparable behavior at cracking and steel yielding load. Failure of the CFRP beams was brittle, while the reference and FeSMA beams exhibited a ductile failure mode with much higher ultimate deflection.

## 6. Practical Implementations

**Figure 14:**
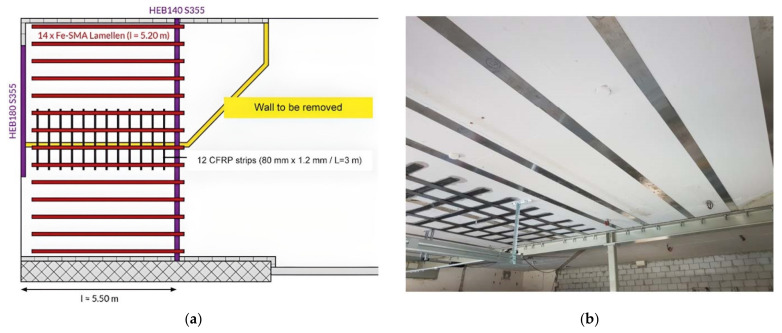
The strengthening of the slab in carpentry in Switzerland: (**a**) scheme of strengthening (republished from [19]); (**b**) view after strengthening (republished from [79]).

The first flexural strengthening strips of real structure with prestressed FeSMA happened in 2017 in Villigen, Switzerland [19,79]. The additional reinforcement was needed since the static system was changed by the removal of load bearing wall. To ensure minimal loss of room height the combination of three strengthening methods was used. The 24 cm thick slab was strengthened by a steel girder installed between two supporting walls and 14 prestressed FeSMA strips applied in the perpendicular directions. Moreover, the lack of overlap of lower steel reinforcement perpendicular to the removed wall was covered by applications of 12 CFRP strips. The scheme of strengthening and finished strengthening project are presented in Figure 14. The FeSMA strips were anchored with nails using a direct fastening system and activated by resistive heating. This heating method caused the activation process is carried out simultaneously through the whole FeSMA strip. It should be mentioned that the location of the internal steel reinforcement of the slab near the end anchorage should be verified to prevent direct contact and possible short circuit.

**Figure 15:**
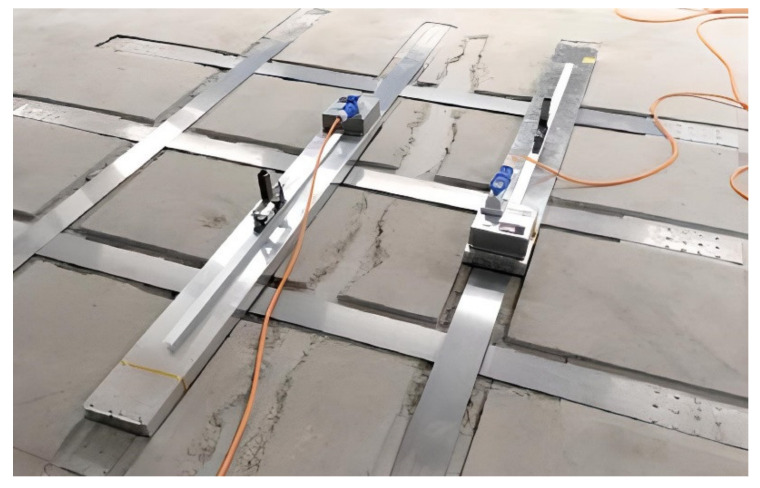
Activation process using infrared heating devices (republished from [79]).

Another possibility of activation FeSMA strips is infrared heating, which was used during strengthening a slab in Arrau, Switzerland [79]. A new column was placed and hence the tensile steel reinforcement of the slab in the negative moment area was insufficient. The FeSMA strips were inserted into grooves to provide an even concrete surface after retrofitting. After activation, the strips were covered with a bonding coat and then the grooves were grouting with a cementitious mortar. The activation process using infrared heating devices does not require a high-power supply as resistive heating (see Figure 15). However, since the infrared heating device is usually shorter than the SMA strip, the activation process takes more time and is carried out sequentially which causes a wide temperature variation in the strip. Strieder et al. [136] indicated that sequentially heating may slightly influence the prestressing level. Further research that compares different heating methods is highly recommended.

**Figure 16:**
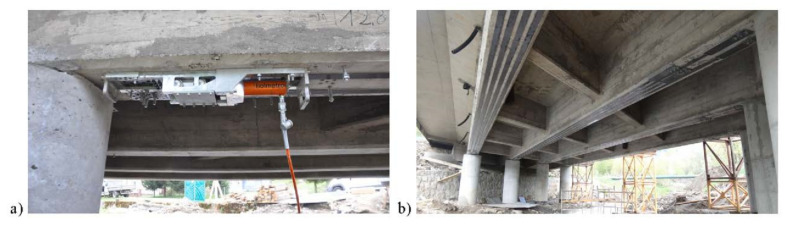
Strengthening of the bridge in Komańcza, republished from [11]: (**a**) prestressing the CFRP strip; (**b**) view of the bridge after strengthening.

Modernization of reinforced concrete bridge in Komańcza, Poland was required due to its deterioration and narrow roadway [11,44]. The deck had to be widened from 8.9 m to 11.2 m. To increase the load capacity of the bridge, the bridge girders were strengthened using prestressed CFRP strips in 2015. The 60 × 1.4 mm strips were applied to the bottom surfaces of beams in mid span and the top surface of the deck over the bridge supports. The bridge was not close to traffic during modernization, hence the strengthening took place in three stages. Firstly, the road surface was milled, the waterproofing was removed, and the strips were installed to the top surface on one half of the deck. Then the bottom surface of the girders was prepared by grinding and the strips were applied to the girders in the main span. Finally, the traffic was redirected and the strips were bonded to the top surface of the second half of the bridge deck. The prestressing force of each strip was estimated as 75 kN. The strip application and view of the bridge after strengthening are presented in Figure 16. The effect of strengthening was verified by comparison strains (of concrete, steel and CFRP strips) and deflections in the main span before (between stages 1 and 2) and after strengthening of the girders. The test load was applied by two 38 tons trucks. The results revealed that the deflections before and after strengthening were almost the same. It was expected due to the small cross section area of the strips in comparison with beam cross section and the small test load that induced relatively low internal forces. This additional load induced the average stresses of 16.5 MPa in the CFRP strips. However, the 23% average reduction of stresses in the tensile steel reinforcement was observed after strengthening that confirmed the effectiveness of the strengthening.

**Figure 17:**
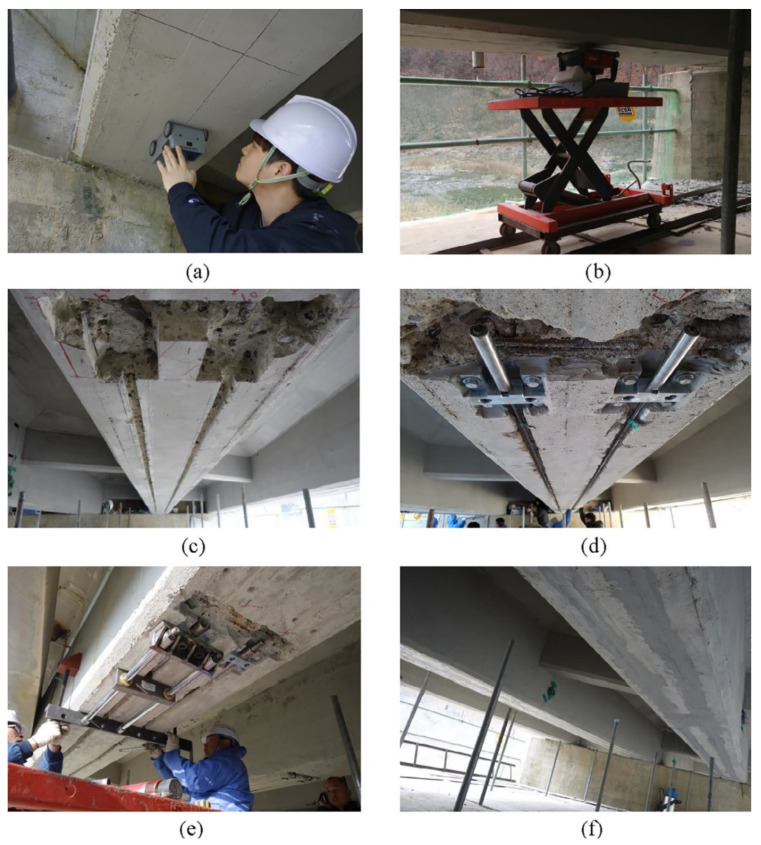
Process of bridge strengthening: (**a**) inspection to identify the thickness of the covering and the location of the steel reinforcement; (**b**) cutting the grooves; (**c**) view of the grooves; (**d**) installation of the anchorage and the CFRP bars.; (**e**) applying prestressing; (**f**) filling the grooves with an epoxy. Reprinted with permission from ref. [143]. Copyright 2018 Elsevier.

The NSM technique was used to strengthen a four-span bridge in Gyeonggi-do, South Korea [143]. The bridge was supported by RC girders. Each girder was strengthened by two CFRP bars. Prestress level of the bar was approximately equal to 100 kN. The prestressing procedure is presented in Figure 17. First of all, the cover thickness and location of steel reinforcement had to be identified. Then, two grooves in each girder were cut using a portable saw-cut machine which was rolled on a trolley along the girder length. In the next stage, steel anchorages were mounted in the cavities between the steel reinforcement of the girders to avoid structural damage. Then, the installation of CFRP bars by threading through the anchor blocks took place. Next, the prestressing force was applied by a hydraulic jack to reach the planned value. After adjusting the fastening nut to hold the force, the jacking device was removed. Finally, epoxy was used to fill all of the grooves and cavities. Before and after strengthening, static (with truck weighing 324 kN placed at the mid span) and dynamic (with the same truck moving 50 km/h) tests were carried out. The results revealed that the maximum displacement of the girders after strengthening decreased from 0.48 to 0.35 mm and from 0.51 to 0.40 mm during static and dynamic tests, respectively. The reduction in strains of steel bars was observed in both tests. The strains decreased by 32 and 26% for static and dynamic loading, respectively. Obtained results confirmed the effectiveness of strengthening to improve the serviceability limit state.

## 7. Conclusions

In this paper, current state-of-the-art developments in terms of prestressed concrete structures using SMA or CFRP reinforcement is presented. Both materials are compared in terms of the material level considering tensile behavior, durability aspects and effects of elevated temperature. Moreover, the prestressing methods and anchorage systems are briefly described. Additional item concerns previous studies considering both experiments and numerical analysis and summary of CFRP and SMA strengthened RC members. The following conclusion can be drawn:

1. The strengthening of concrete structures with prestressed CFRP and SMA materials is significantly efficient in serviceability states, including higher cracking load and reduced deflections. The effectiveness of flexural strengthening was confirmed both in laboratory tests and practical implementations.
2. Prestressing using SMAs can be an interesting alternative to prestressing with CFRP materials, especially due to the future price reduction of SMAs.
3. SMA materials can be used as prestressed near surface mounted or externally applied reinforcement. In both cases, they are much easier to apply anchors and activate SMA compared to CFRP materials, which usually require specialized equipment. The FeSMAs are activated by heating to the temperature, which is usually in the range of 130–200 °C.
4. The activation process can be performed using different methods: resistive heating, infrared heating or heating using a gas burner. The prestressing level may be affected by the activation method, however further research on this topic is recommended.
5. The prestressing level of SMAs depends on the type of the alloy, activation temperature and the initial prestraining level. In the presented state of the art, the shape recovery stress was in the range of 130–450 MPa, which resulted in 2–4 times lower prestressing forces than by using CFRP reinforcement. Hence, the flexural capacity of the beams strengthened with CFRP bars and strips is higher than SMAs.
6. The most common failure mode for beams strengthened with CFRP reinforcement was CFRP rupture or CFRP delamination, while the beams strengthened with FeSMAs exhibited usually a failure due to concrete crushing after the steel yielding. A proper design of strengthening using CFRP reinforcement is needed to utilize their high tensile strength and to prevent the brittle failure mode.
7. The behavior of SMA is very ductile that allows the RC members to obtain much higher deflections. The ductility index for all beams strengthened with SMA was much higher than for CFRP strengthened beams.
8. Two important factors should be considered during the design of strengthening using prestressed memory steel. In first the elasticity modulus depends on the actual state of the alloy since the initial value is twice higher than the value after activation. The latter is appropriate for design purposes. Secondly, the compressive strains in concrete should be limited to 2–2.5‰ as the SMA reinforcement behaves similarly as the external prestressed unbonded tendon.
9. The thermal compatibility with concrete is much better in the case of SMAs, since they have a slightly higher coefficient of thermal expansion than concrete. As the coefficient of thermal expansion of CFRP is close to 0 or even negative the stress changes occur along with the bonded CFRP reinforcements due to the temperature changes.
10. The memory steel exhibits much better performance compared to CFRP when is subjected to elevated temperatures. Additionally, heating and toxic gases are released due to thermal decompositions of the FRP matrix when exposed to a temperature of 300–400 °C. Hence, it is necessary to provide proper fire protection not only due to the strength degradation but also due to the smoke toxicity.
11. The prestress losses in the RC members strengthened with prestressed CFRP materials occurs in the first 100 h and a further decrease in prestressing is negligible, while the memory steel is affected by long-term creep and stress relaxation. The current studies estimate that the prestress losses due to relaxation are about 15%. However, they might be retrieved by second or multiple activations.
12. The CFRPs are fully corrosion resistant due to their non-metallic structure, while SMAs are susceptible to corrosion and that should be taken into account in long-term durability. Memory steel is especially sensitive to chloride ions, which intensifies the pitting corrosion. Hence, additional corrosion protection should be applied for structures in aggressive environments.
13. On-site tests confirmed the practicality of flexural strengthening using prestressed CFRPs for RC bridge structures under dynamic load conditions.
14. Strengthening with FeSMA materials can be combined with other strengthening techniques (e.g., non-prestressed CFRP strips) to ensure maximum gains in serviceability and ultimate limit states.

Future research is necessary to develop the current knowledge of strengthening RC structures with prestressed SMA materials. The following recommendations should be taken into future research:

1. The prestress losses and long-term behavior of SMAs used for prestressing of RC structures.
2. The prestressing losses due to the slippage at anchorages should be analyzed.
3. Developing more precise methods of determining prestressing level in memory steel after its application to RC structure.
4. Effects of different heating methods on the prestressing level in SMA materials.
5. The possibility of multiple activations should be developed.
6. The behavior of SMA in chemical environments considering effects on recovery stress or bonding behavior should be investigated in more detail.
7. Feasibility of SMA materials for flexural strengthening of RC structures under dynamic load conditions.
8. Effectiveness of corrosion protection applied to SMA materials.
9. Design guidelines for prestressing with SMA should be organized.
10. Comparison of prestressing techniques using SMA and unbonded CFRP reinforcement with end-anchorages.

## Figures and Tables

**Table 1:** Characteristics of FeSMAs [18,26,83], prefabricated CFRP strips [84] and steel.

Material	E_SMA/_E_f_/E_s_ (GPa)	f_SMA/_f_f_/f_s_ (MPa)	ε _SMA/_ε_f_/ε_s_ (%)
FeSMAs	160–170	680–1000	16–50
Low modulus CFRP strips	170	2800	1.6
High modulus CFRP strips	300	1300	0.5
Steel	200	600	25

E_SMA,_ E_f,_ E_s_: elasticity modulus of SMA and CFRP materials and steel; f_SMA,_ f_f,_ f_s_: tensile strength of SMA and CFRP materials and steel; ε_SMA,_ ε_f,_ ε_s_: ultimate tensile strain of SMA and CFRP materials and steel.

**Table 2:** Electrochemical parameters of various types of FeSMAs and steel.

Ref.	Material	Solution	Chloride in Solution	pH	Exchange Current Density i_corr_ [μA/cm^2^]
[104]	Fe-25Mn-6Si-5Cr Fe-26Mn-6Si-5Cr-0.16La Fe-25Mn-6Si-5Cr-0.30La 18-8 stainless steel	3.5 wt. % NaCl aqueous solution	Yes	N/I	671.82 7.20 58.31 3.17
[102]	Fe-17Mn-5Si-10Cr-4Ni-0.74V S500 steel Fe-17Mn-5Si-10Cr-4Ni-0.74V S500 steel	15 mM NaHCO_3_ + 5 mM Na_2_CO_3_ 1.2 M CH_3_COOH + 3.74 M CH_3_COONa	No	8.4 8.4 4.5 4.5	0.67 3.30 0.57 77.00
[106]	Fe-14Mn-4Si-9Cr-4Ni Fe-14Mn-4Si-9Cr-4Ni-0.18Ce Fe-14Mn-4Si-9Cr-4Ni-0.42Ce Fe-14Mn-4Si-9Cr-4Ni-0.96Ce	10 mM Na_2_SO_4_ + 400 mM KOH + 1 mM Ca(OH)_2_	No	13	0.27 0.28 0.34 0.33
[106]	Fe-14Mn-4Si-9Cr-4Ni Fe-14Mn-4Si-9Cr-4Ni-0.18Ce Fe-14Mn-4Si-9Cr-4Ni-0.42Ce Fe-14Mn-4Si-9Cr-4Ni-0.96Ce	10 mM Na_2_SO_4_ + 400 mM KOH + 1 mM Ca(OH)_2_ + 0.6 M NaCl	Yes	13	0.36 0.28 1.99 1.14
[110]	Fe-16Mn-5Si-4Ni-5Cr-0.3C-1Ti S400 Steel	3.5 wt.% NaCl solution with various pH values, adjusted by CaO	Yes	7 9 11 13 7 9 11 13	4.70 3.20 1.60 0.38 17.00 4.30 2.20 0.77

**Table 3:** Experimental and numerical tests of RC members strengthened with FeSMA and CFRP materials.

Refs.	Beam ID	Analysis Type	A_c_ [mm × mm]	f_c_ [MPa]	A_s,t_ [mm]	A_s,c_ [mm]	f_y,s_ [MPa]	PS	ST	A_Str_ [mm × mm]	f_u,SMA_/f_u,CFRP_ [MPa]	E_SMA_/E_CFRP_ [GPa]	Pres [MPa]	End Anch.	Adhesive	Load Type	Fail. Mode
[19]	Ref_Beam CFRP_Beam FeSMA_B1 FeSMA_B2	Exp	150 × 500	33.8	3 #10	3 #10	518	- EBR EAR EAR	- 1 S 1 S 1 S	- 50 × 1.4 100 × 1.5 100 × 1.5	- 2800 981 981	- 170 80 * 80 *	0 0 440 410	- - EA EA	- epoxy - -	4PBT	CC SD CC CC
[128]	Ref_Beam CFRP_Beam FeSMA_Beam	Exp	305 × 150	38	2#16	2#11.3	458	- NSM NSM	- 1 S 1 B	- N/A #14.3	- 2068 826	- N/A N/A	0 695 131	- N/A EA	- N/A epoxy	4PBT	CC CC CC
[17]	B1_Ref B2_FeSMA B3_FeSMA B4_FeSMA B5_CFRP B6_FeSMA	Exp	150 × 250	53.4	2 #8	2 #8	508	- NSM NSM NSM NSM NSM	- 2 S 2 S 2 S 1 S 2 S	- 20 × 1.7 20 × 1.7 20 × 1.7 N/A 20 × 1.7	- N/A N/A N/A 2683 N/A	- N/A N/A N/A 150 N/A	0 0 190 193 0 213	- - - - - -	- CBM CBM CBM epoxy CBM	4PBT	CC CC CC SR SR SR
[129]	FeSMA CFRP	Num	150 × 250	58.3	2 #8	2 #8	508	NSM	2 S 1 S	20 × 1.7 N/A	N/A 2683	N/A 150	190 0	- -	CBM N/A	4PBT	CC SR
[130]	0CFRP-32 20CFRP-32 30CFRP-32 40CFRP-32 0FeSMA-32 20FeSMA-32 30FeSMA-32 40FeSMA-32	Num	300 × 200	61	2 #16	2 #16	496	NSM	1 R	10 × 10	2800 2800 2800 2800 1000 1000 1000 1000	160 160 160 160 160 160 160 160	0 560 840 1120 0 200 300 400	- - - - - - - -	epoxy epoxy epoxy epoxy epoxy epoxy epoxy epoxy	4PBT	RR RR RR RR CC CC CC CC
[131]	Ref_Beam CFRP_B1 FeSMA_B1 CFRP_B2 FeSMA_B2	Num	400 × 200	40	3 #16	2 # 11.3	475	- NSM NSM NSM NSM	- 1 B 7 S 1 B 14 S	- #9 10 × 1.5 #9 10 × 1.5	- 2167 990 2167 990	- 130 N/A 130 N/A	0 440 260 880 260	- N/A EA N/A EA	- epoxy N/A epoxy N/A	4PBT	CC BR CC BR CC

Analysis type: Exp, experimental tests and Num, numerical analysis; A_c_, dimensions of concrete cross section [height × width]; f_c_, concrete compressive strength; A_s,t_, tensile steel reinforcement [number of bars #diameter]; A_s,c_, compressivee steel reinforcement [number of bars #diameter]; f_y,s_, steel yielding strength; PS, prestressing system: EBR, externally bonded reinforcement, EAR, externally applied reinforcement, NSM, near-surface mounted reinforcement and shot is reinforcement embedded in a shotcrete layer; ST, strengthening type: S, acronym of strip, B, acronym of bar, R, acronym of rod; A_Str_, dimensions of SMA/CFRP cross section [width × depth or #diameter]; f_u,SMA_/f_CFRP_, ultimate strength of SMA/CFRP; E_SMA_/E_CFRP_, young modulus of SMA/CFRP: * means that young modulus after activation was given; End anch., end anchorage (acronym EA means that reinforcement was anchored of its end); Adhesive: CBM, cement based mortar, epoxy is epoxy adhesive, shot is a shotcrete layer; Load type: 4PBT, four point bending test; FAT, a fatigue test; CAN, test with force on the slab cantilever; Fail. mode, failure mode: CC, concrete crushing after steel yielding; SD, strip debonding; ICD, interfacial debonding; CCS, concrete cover separation; AF, anchorage failure; SR, BR, and RR, strip, bar or rod rupture, respectively; N/A, data not available.

**Table 4:** Experimental and numerical tests of RC members strengthened with FeSMA materials.

Refs.	Beam ID	Analysis Type	A_c_ [mm × mm]	f_c_ [MPa]	A_s,t_ [mm]	A_s,c_ [mm]	f_y,s_ [MPa]	PS	ST	A_SMA_ [mm × mm]	f_u,SMA_ [MPa]	E_SMA_ [GPa]	Prestress [MPa]	End Anch.	Adhesive	Load Type	Fail. Mode
[82]	Ref_Beam FeSMA_Beam	Exp	305 × 150	39.1	2 #16	2 # 11.3	400	- NSM	- 1 B	- #14.3	- 780	- N/A	0 N/A	- EA	- CBM	4PBT	CC CC
[132]	C-B SMA-B	Num	305 × 150	39.1	2 #16	2 # 11.3	440	- NSM	- 1 B	- #14.3	- 820	- N/A	0 N/A	- EA	- CBM	4PBT	CC CC
[133]	Ref_Beam FeSMA_Beam	Exp	305 × 150	35.3	2 #16	2 # 11.3	505	- NSM	- 1 B	- #14.3	- 820	- N/A	0 N/A	- N/A	- N/A	FAT	N/A N/A
[21]	B-C B-SMA-0 B-SMA-1	Exp	305 × 150	39.9 39.9 41	2 #16	2 # 11.3	458 458 505	- NSM NSM	- 1 B 1 B	- #14.3 #14.3	- N/A N/A	- N/A N/A	0 130 200	- EA EA	- epoxy CBM	4PBT	CC CC CC
[134]	R-C R-SMA E-C E-SMA	Exp	305 × 150	43 43 36.8 38.4	2 #16	2 # 11.3	#16—451 #11.3—440	- NSM - NSM	- 1 B - 1 B	- #14.3 - #14.3	- 780 - 780	- N/A - N/A	0 130 0 200	- EA - EA	- CBM - CBM	4PBT	CC CC CC CC
[135]	Ref_Beam FeSMA_B1 FeSMA_B2	Num	150 × 250	39.1	2 #16	2 # 11.3	508	- NSM NSM	- 2 S 2 S	- 20 × 1.7 20 × 1.7	- N/A N/A	- N/A N/A	0 0 190	- - -	- CBM CBM	4PBT	CC CC CC
[22]	B-C B-SMA-0 B-SMA-1 B-SMA-2	Exp	400 × 200	40	3 #16	2 #11.3	#16—410 #11.3—474	- NSM NSM NSM	- 5 S 5 S 7 S	- 15.8 × 1.5 15.8 × 1.5 15.8 × 1.5	- 990 990 990	- 116 116 116	0 0 274 274	- EA EA EA	- epoxy epoxy epoxy	4PBT	CC CC CC CC
[23]	B7_FeSMA B8_FeSMA	Exp	150 × 250	53.4	2 #8	2 #8	508	NSM NSM	2 S 2 S	20 × 1.7 20 × 1.7	~760 ~760	160 160	0 ~200	- -	CBM CBM	4PBT	CC CC
[20]	B1_Ref B9_Steel B10_FeSMA B11_FeSMA	Exp	140 × 250 160 × 250	59	2 #8	2 #8	508	- shot shot shot	- 2 B 2 B 2 B	- #8 #8 #8	- N/A N/A N/A	- N/A N/A N/A	0 0 285 307	- - - -	- shot shot shot	4PBT	CC CC CC CC
[24]	1-ref 2-CR-act 3-NSM 4-NSM	Exp	230 × 100	64.3 64.1 69.2 62.5	5 #12	5 #12	513	- shot NSM NSM	- 5 B 5 B 5 B	- #11.5 #11.5 #11.5	- N/A N/A N/A	- N/A N/A N/A	0 322 0 307	- - - -	- shot CBM CBM	CAN	CC CC CC CC
[136]	Ref_Beam Beam_steel SMA_1 SMA_2	Exp	150 × 250	59	2#8 + 2#12	2#8	500	- NSM NSM NSM	- 1 S 1 S 1 S	- 50 × 2.3 50 × 2.3 50 × 2.3	- N/A N/A N/A	- N/A N/A N/A	0 0 278 347	- N/A EA EA	- - - -	4PBT	N/A N/A N/A N/A
[137]	B9_Steel B10_FeSMA	Num	160 × 250	59	2 #8	2 #8	508	shot shot	2 B 2 B	#8 #8	N/A N/A	N/A N/A	0 0.100.300.400	- -	shot shot	4PBT	N/A N/A
	Girder_ref G1 G2 G3 G4 G5	Num	1000 × 300	64.6	5 #10.5	-	1660	shot	- 3 B 3 B 3 B 3 B 3 B	- #5 #6 #7 #8 #9.2	- N/A N/A N/A N/A N/A	- N/A N/A N/A N/A N/A	- 0.300 0.300 0.300 0.300 0.300	- - - - - -	- shot shot shot shot shot	4PBT	N/A N/A N/A N/A N/A N/A

**Table 5:** Experimental and numerical tests of RC members strengthened with CFRP materials.

Refs.	Beam ID	Analysis type	A_c_ [mm × mm]	f_c_ [MPa]	A_s,t_ [mm]	A_s,c_ [mm]	f_y,s_ [MPa]	PS	ST	A_CFRP_ [mm × mm]	f_u,CFRP_ [MPa]	E_CFRP_ [Gpa]	Prestress [MPa]	End Anch.	Adhesive	Load Type	Fail. Mode
[58]	Contro l0% Prestress. 40% Prestress. 60% Prestress.	Exp	254 × 152	45	2 #16	2 # 11.3	440	- NSM NSM NSM	- 1 B 1 B 1 B	- #9.5 #9.5 #9.5	- 1970 1970 1970	- 136 136 136	0 0 788 1182	- - - -	- epoxy epoxy epoxy	4PBT	CC CC BR BR
[59]	B00 B2–0% B2–20% B2–40% B2–60%	Exp	400 × 200	40	2 #16	2 # 11.3	#16—475 #11.3—500	- NSM NSM NSM NSM	- 1 B 1 B 1 B 1 B	- #9 #9 #9 #9	- 2068 2068 2068 2068	- 124 124 124 124	0 0 414 827 1241	- EA EA EA EA	- epoxy epoxy epoxy epoxy	4PBT	N/A BR BR BR BR
[138]	SREF S2L-0 S2L-20 S2L-40	Exp	120 × 600	15	4 #8	3 #6	#8—556 #6—528	- NSM NSM NSM	- 2 S 2 S 2 S	- 20 × 1.4 20 × 1.4 20 × 1.4	- 2770 2770 2770	- 176 176 176	0 0 554 1108	- - - -	- epoxy epoxy epoxy	4PBT	CC SR SR SR
[139]	US RS-2N20 PRS-EB PRS-2N20 PRS-1N45 PRS-2N20-BL PRS-2N20-A	Exp	350 × 150	37.6 32.1 26.6 30.4 35.5 60.8 59.5	2 #16	2 #22	N/A	- NSM EBR NSM NSM NSM NSM	- 2 S 1 S 2 S 1 S 2 S 2 S	- 16 × 2.0 50 × 1.2 16 × 2.0 16 × 4.5 16 × 2.0 16 × 2.0	- 2068 3100 2068 2068 2068 2068	- 131 165 131 131 131 131	0 0 1000 1000 1000 1000 1000	- - EA - - - EA	- epoxy epoxy epoxy epoxy epoxy epoxy	4PBT	CC ICD ICD CCS ICD SR SR
[140]	Control B-40-0-4800 UB-40-0-4800 B-20-0-4800 B-40-30-4800 B-40-60-4800	Exp	600 × 400	40 40 40 20 40 40	3 #19	3 #22	N/A	- NSM NSM NSM NSM NSM	- 1 B 1 B 1 B 1 B 1 B	- #10 #10 #10 #10 #10	- 2081 2081 2081 2081 2081	- 170 170 170 170 170	0 1040 1040 1040 1040 1040	- EA EA EA EA EA	- epoxy - epoxy epoxy epoxy	4PBT	CC BR AF BR BR BR
[141]	B1 B2 B3 B4 B5 B6	Exp	420 × 500	62	3 #25	3 #25	522	- EBR EBR EBR EBR EBR	- 2 S 2 S 2 S 2 S 2 S	- 60 × 1.4 60 × 1.4 60 × 1.4 60 × 1.4 60 × 1.4	- 3200 3200 3200 3200 3200	- 160 160 160 160 160	0 0 0 960 1280 1600	- - EA EA EA EA	- epoxy epoxy epoxy epoxy epoxy	4PBT	CC ICD ICD SR SR AF
[99]	B12-asp B12-sp B12-asp-e B12-sp-e B12-a B16-asp B16-asp-e	Exp	220 × 500	35.3 33.8 46.7 44.0 50.3 52.4 60.3	4#12 4#12 4#12 4#12 4#12 4#16 4#16	4#8	511 511 540 540 540 595 595	EBR EAR EBR EAR EBR EBR EBR	1 S 1 S 1 S 1 S 1 S 1 S 1 S	100 × 1.2 100 × 1.2 100 × 1.2 100 × 1.2 100 × 1.2 100 × 1.2 100 × 1.2	2857 2857 2857 2857 2857 2857 2857	175 175 175 175 175 175 175	900 796 822 762 885 831 840	EA EA EA EA - EA EA	epoxy - epoxy - epoxy epoxy epoxy	6PBT	ICD CC ICD AF SD ICD ICD
[72]	NFCB1 NFCBW2 PRCB1-0R PRCB1-2R PRCB1-4R PRCB1-6R PRCB1-7R	Exp	300 × 200	16.4—20.7	3 #10	3 #13	420	EBR EBR EBR EBR EBR EBR EBR	1 S 2 S 1 S 1 S 1 S 1 S 1 S	50 × 1.4 50 × 1.4 50 × 1.4 50 × 1.4 50 × 1.4 50 × 1.4 50 × 1.4	2161 2161 2161 2161 2161 2161 2161	165 165 165 165 165 165 165	0 0 0 432 864 1296 1512	- - EA EA EA EA EA	epoxy epoxy epoxy epoxy epoxy epoxy epoxy	3PBT	SD SD ICD ICD ICD ICD SR

**Table 6:** Selected results from the previous studies.

Refs.	Beam ID	Prestress [MPa]	P_cr_ [kN]	Δ_cr_ [mm]	P_y_ [kN]	Δ_y_ [mm]	P_u_ [kN]	Δ_u_ [mm]	I_D_ = Δ_u_/Δ_y_ [-]
[19]	Ref_Beam	0	1.0	0.8	5.6	40.0	7.9	145.0	3.63
	CFRP_Beam	0	2.0	1.2	11.3	49.0	14.9	75.0	1.53
	FeSMA_B1	440	3.4	3.2	11.7	39.0	17.1	105.0	2.69
	FeSMA_B2	410	5.6	3.2	12.8	42.0	15.4	135.0	3.21
[128]	Ref_Beam	0	19.0	N/A	104.0	5.3	124.0	18.0	3.40
	CFRP_Beam	695	43.0	N/A	141.0	5.3	178.1	16.5	3.11
	FeSMA_Beam	131	40.0	N/A	126.9	5.8	165.4	27.5	4.74
[17]	B1_Ref	0	2.0	N/A	N/A	N/A	9.8	48.7	N/A
	B2_FeSMA	0	2.5	N/A	N/A	N/A	16.8	70.6	N/A
	B3_FeSMA	190	4.5	N/A	N/A	N/A	16.9	56.9	N/A
	B4_FeSMA	193	4.7	N/A	N/A	N/A	16.8	51.1	N/A
	B5_CFRP	0	2.4	N/A	N/A	N/A	22.9	55.9	N/A
	B6_FeSMA	213	4.2	N/A	N/A	N/A	16.4	52.0	N/A
[129]	FeSMA	190	N/A	N/A	~13.0	N/A	~16.6	~83.0	N/A
	CFRP	0	N/A	N/A	~13.0	N/A	~22.5	~60.0	N/A
[130]	0CFRP-32	0	15.2	2.0	93.9	20.5	127.6	45.8	2.24
	20CFRP-32	560	27.5	2.1	110.7	20.0	130.4	32.0	1.60
	30CFRP-32	840	28.6	2.0	120.5	19.6	138.3	31.0	1.58
	40CFRP-32	1120	29.3	2.0	128.5	20.2	131.5	26.8	1.32
	0FeSMA-32	0	13.8	2.5	83.2	29.3	105.7	72.2	2.75
	20FeSMA-32	200	22.1	2.6	94.3	27.4	108.2	60.5	2.48
	30FeSMA-32	300	23.6	2.5	101.7	25.9	111.3	58.4	2.45
	40FeSMA-32	400	24.5	2.4	109.5	24.2	114.7	54.6	2.38
[131]	Ref_Beam	0	32.0	3.3	82.4	22.5	90.7	157.8	7.01
	CFRP_B1	440	52.6	4.5	119.0	26.6	160.9	91.9	3.45
	FeSMA_B1	260	53.6	4.2	120.0	24.3	145.0	163.5	6.72
	CFRP_B2	880	65.5	4.3	136.0	24.3	162.8	53.0	2.18
	FeSMA_B2	260	65.0	4.0	153.0	25.8	188.9	139.6	5.40

P_cr_, P_y_, and P_u_ are cracking, yielding and ultimate loads, respectively; Δ_cr_, Δ_y_, and Δ_u_ are deflection at cracking, yielding and ultimate, respectively; I_D_ is a ductility index, calculated as deflection at ultimate load divided by deflection at yielding load; N/A means that the data are not available.

## Data Availability

The data presented in this study are available on request from the corresponding author.
